# Association of current smoking with airway inflammation in chronic obstructive pulmonary disease and asymptomatic smokers

**DOI:** 10.1186/1465-9921-6-38

**Published:** 2005-04-25

**Authors:** Brigitte WM Willemse, Nick HT ten Hacken, Bea Rutgers, Dirkje S Postma, Wim Timens

**Affiliations:** 1 Department of Pathology, University Medical Centre Groningen, Groningen, The Netherlands; 2 Department of Pulmonology, University Medical Centre Groningen, Groningen, The Netherlands

**Keywords:** current smoking, bronchial biopsies, sputum

## Abstract

**Background:**

Inflammation in the airways and lung parenchyma underlies fixed airway obstruction in chronic obstructive pulmonary disease. The exact role of smoking as promoting factor of inflammation in chronic obstructive pulmonary disease is not clear, partly because studies often do not distinguish between current and ex-smokers.

**Methods:**

We investigated airway inflammation in sputum and bronchial biopsies of 34 smokers with chronic obstructive pulmonary disease (9 Global initiative for Chronic Obstructive Lung Disease stage 0, 9 stage I, 10 stage II and 6 stage III) and 26 asymptomatic smokers, and its relationship with past and present smoking habits and airway obstruction.

**Results:**

Neutrophil percentage, interleukin-8 and eosinophilic-cationic-protein levels in sputum were higher in chronic obstructive pulmonary disease (stage I-III) than asymptomatic smokers. Inflammatory cell numbers in bronchial biopsies were similar in both groups. Current smoking correlated positively with macrophages: in bronchial biopsies in both groups, and in sputum in chronic obstructive pulmonary disease. Pack-years smoking correlated positively with biopsy macrophages only in chronic obstructive pulmonary disease.

**Conclusion:**

Inflammatory effects of current smoking may mask the underlying ongoing inflammatory process pertinent to chronic obstructive pulmonary disease. This may have implications for future studies, which should avoid including mixed populations of smokers and ex-smokers.

## Background

Chronic obstructive pulmonary disease (COPD) is one of the most important causes of death and its prevalence is still increasing [1]. The major risk factor in the development and progression of COPD is cigarette smoking. COPD is characterised by fixed airway obstruction and respiratory symptoms, i.e. chronic cough, sputum production and dyspnoea. COPD patients have, just like so called healthy smokers, an inflammatory reaction involving the entire tracheobronchial tree [2,3].

As compared to healthy non-smokers the degree of airway inflammation seems higher in COPD patients. For example, higher numbers of CD8 positive T-cells, macrophages, neutrophils, and mast cells, both in central and peripheral airways have been found in COPD patients, irrespective whether these patients were current smokers or ex-smokers [4-10]. In addition, the percentage of neutrophils and IL-8 levels in sputum and broncho-alveolar lavage of COPD patients were higher [7,11-16]. As compared to healthy smokers, the differences with COPD patients are less clear cut. For example, higher numbers of neutrophils, macrophages and CD8 positive T-cells in the peripheral airways of COPD patients were found as compared to smokers [10,17-19], whereas others did not [10,19,20]. Two studies showed a higher percentage of neutrophils and higher IL-8 levels in broncho-alveolar lavage of COPD patients [13,21], whereas Linden et al. found no differences [7]. A few studies showed higher numbers of neutrophils [22], CD3, CD4[23] CD8 positive T-cells [23,24] in bronchial biopsies, whereas other studies found no differences in neutrophils [24], CD3, CD4 [22,24] and CD8 positive T-cels [22], macrophages, eosinophils and mast cells [22,24]. In conclusion, COPD patients have a higher degree of airway inflammation compared to non-smokers, however it remains unclear whether this is also true comparing COPD patients with so called healthy smokers.

Definite conclusions about the exact role of cigarette smoking in COPD are difficult to draw for a number of reasons. First, most studies investigated smokers combined with ex-smokers. Second, many studies investigated COPD patients combined with patients with chronic bronchitis. Third, many studies investigated only one aspect of inflammation, or only one compartment (sputum, broncho-alveolar lavage, bronchial biopsies, peripheral airways), which may be insufficient to obtain a full view. Fourth, remodelling in COPD may itself generate and maintain an inflammatory process, independent of cigarette smoking [25].

In order to elucidate the role of smoking on inflammation in COPD we have investigated airway inflammation in sputum and bronchial biopsies of asymptomatic smokers and smokers with COPD. Furthermore, we assessed whether airway inflammation is related to the number of cigarettes smoked per day, to pack-years smoking and to severity of airway obstruction.

## Methods

### Subjects

Subjects were recruited from the pulmonary outpatient clinic of the Groningen University Hospital and by advertisements in local newspapers. 34 smokers with COPD and 26 smokers without COPD were included according to the ERS criteria [26]. Smokers with COPD had chronic cough and sputum production for at least 3 months for 2 successive years, and an forced expiratory volume in one second (FEV_1_)/ vital capacity (VC) ≤ 88% of predicted for males and ≤ 89% of predicted for females. Asymptomatic smokers without COPD had no chronic respiratory symptoms, and FEV_1_/VC >88% of predicted for males and >89% of predicted for females and an FEV_1 _>85% of predicted. To detect respiratory symptoms to delineate the group of symptomatic smokers without COPD we used the questions about respiratory symptoms and smoking from the Dutch version of the British Medical Research Council's standardised questionnaire [27]. These data were collected by interviewing the participants at the first visit. All participants had to meet the following criteria: age between 45–75 years, minimum of 10 pack-years smoking, actual smoking ≥ 10 cigarettes per day, reversibility to salbutamol < 9% of the predicted FEV_1_, no use of inhaled or oral corticosteroids in the previous 6 months, no atopy (no positive skin prick test for 10 common aeroallergens and serum total IgE < 200 IU), no respiratory tract infection 1 month prior to the study. After inclusion, subjects were categorized according to the Global Initiative for Chronic Obstructive Lung Disease, GOLD stage 0-IV [28]. GOLD stage 0 (symptomatic smokers): 'at risk' with normal spirometry but chronic symptoms (cough, sputum production); GOLD stages I-IV: FEV_1_/FVC post bronchodilator (post BD) < 70% and GOLD stage I: FEV_1 _post BD ≥ 80% predicted; GOLD stage II: 50% ≤ FEV_1 _post BD < 80% predicted; GOLD stage III: 30% ≤ FEV_1 _post BD <50% predicted and GOLD stage IV: 30% ≤ FEV_1 _post BD or FEV_1_<50% predicted plus respiratory failure. Current smoking was confirmed by urinary cotinine levels > 25 ng/ml. Before each measurement subjects were asked not to use long or short-acting β_2 _agonists and/or ipratropium at least 12 hours before the test. The local medical ethics committee approved the study protocol and all subjects gave their written informed consent.

### Study Design

All subjects visited the hospital on 5 separate days, at least one week apart. Lung function tests (flow-volume curves, reversibility, airway conductance), airway hyperresponsiveness (AHR) to methacholine and to adenosine-5'-monophosphate (AMP), and sputum induction (twice) were performed and all subjects underwent bronchoscopy.

### Lung Function

Lung function (FEV_1_, FEV_1_/VC) was measured using dry wedge spirometry (Masterscope, Jaeger, Breda, The Netherlands) according to standardized guidelines [29]. Specific airway conductance (sGaw) was measured by body plethysmography (Masterscope, Jaeger, Breda, The Netherlands). Provocation tests were performed with a 2-minute tidal breathing method adapted from Cockcroft and co-workers [30]. After an initial nebulised saline (0.9%) challenge, subjects inhaled doubling concentrations, ranging from 0.038 to 39.2 mg/ml of methacholine-bromide (Sigma Chemical Co. St Louis, MO) and from 0.04 to 320 mg/ml of AMP (Sigma Chemical Co. St Louis, MO) at 5-minute intervals. Tests were terminated when FEV_1 _had fallen 20% or more from its baseline value (PC_20_).

### Sputum Induction and Sputum Processing

Sputum was induced by inhalation of hypertonic saline aerosol and processed as described previously [31]. Briefly 15 minutes after salbutamol (400 μg) inhalation, hypertonic saline (3%, 4% and 5% w/v) was nebulised and inhaled for each concentration over a period of 7 minutes. Whole sputum samples were processed within 2 hours after termination of the induction. Two sputum cytospin slides were stained with May-Grünwald-Giemsa for differential cell counts. Counting of 600 non-squamous cells in a blinded way by one technician (B.R.). Sputum samples containing > 80% of squamous cells were excluded from analysis as indication of poor cytospin quality. Interleukin 8 (IL-8) concentration was measured using ELISA (CLB, Amsterdam, the Netherlands) and eosinophil cationic protein (ECP) concentration by a fluorenzyme immunoassay (ImmunoCAP ECP, Pharmacia, Uppsala, Sweden).

### Bronchoscopy and biopsy processing

Subjects were not allowed to drink or eat at least 4 hours prior to the bronchoscopy. Smoking was not allowed before the bronchoscopy. On arrival, FEV_1 _was measured before and 15 minutes after 400 μg salbutamol. Hereafter subjects gargled with 5 ml of 2% lidocaine and had 2% lidocaine sprayed on the posterior pharynx, dripped onto the vocal cords and into the trachea, with a maximum dose of 3 mg/kg lidocaine. A flexible fiberoptic bronchoscope (Olympus B1 IT10, Olympus Optical, Tokyo, Japan) was introduced and preferably 6 bronchial biopsies were taken from the subcarinae of the right middle or lower lobe using a fenestrated cup forceps (Olympus FB-21C, Olympus Optical Tokyo, Japan) [32]. Biopsies were collected into sterile PBS on ice. Two biopsies were directly embedded in Tissue Tek (Bayer Corporation, Elkhart, Indiana, USA), snap-frozen in liquid isopentane and stored at -80°C, 4 biopsies were fixed in 4% paraformaldehyde, processed and embedded in paraffin.

Serial sections were cut from the paraffin biopsies with a thickness of 4 μm and stored at room temperature. Selection of morphological optimal tissue was based on a hematoxylin and eosin stained slide. Tissue slides were deparaffinised with xylene (15 min) and dehydrated before staining. Immunohistochemical staining was performed with monoclonal antibodies against: CD3 (A0452, DAKO, Copenhagen, Denmark) CD4 (CD4-368, Novacastra, UK), CD8 (M7103, DAKO), B cells (CD20 L26, M0755, DAKO), mast cell tryptase (AA1, M7052, DAKO), neutrophil elastase (NP57, M0752, DAKO), macrophages (CD68, M0814, DAKO) and secreted form of eosinophilic cationic protein EG2 (Pharmacia Diagnostics, Sweden). Negative controls were obtained by omission of the primary antibody. Slides were pre-treated with 1 mM EDTA buffer pH = 8 (CD4, CD8), 0.1 mM tris-HCL buffer pH = 9.0 (CD20) in the microwave for 8 or 30 minutes respectively or with 1% protease for 30 minutes at room temperature (CD68, NP57, AA1, EG2). CD3 slides were incubated overnight at 80°C with tris/HCL buffer pH = 9.0. All stainings were performed in an automated system using the Dako Autostainer (DAKO, Copenhagen, Denmark), except for CD4 that was done manually.

The dilutions used were: CD3 1:100; CD4 1:25; CD8 1:100; CD68 1:50; EG2 1:200; NE 1:200; AA1 1:100; CD20 1:400. As detection system we used labelled streptavidin-biotin (LSAB+, K0690, DAKO, Copenhagen, Denmark) except for CD4 where the Envision system (K5007, DAKO, Copenhagen, Denmark) was used. 3-amino-9- Ethyl Carbazole (AEC) (K3469, DAKO, Copenhagen, Denmark), or Nova Red (SK4800, Vector, USA) for CD4, was used as a chromogen (substrate) giving a reddish-brown reaction product. Hydrogen peroxide was used for blocking endogenous peroxidase and haematoxylin was used as a counterstain. For each antigen, all slides were stained simultaneously.

For each immunohistochemical staining 2 sections of 2 different bronchial biopsies were quantified by computer-assisted image analysis at magnification of 200× (Qwin, Leica Microsystems Imaging Solutions Ltd, Cambridge, UK). Automated image analysis to quantify immunopositivity was performed using the next algorithm: first the intensity of the positive area (cells) was appointed in each biopsy by the observer, followed by the intensity of the total area of the biopsy, based on the red-green-blue (RGB) color model [33,34]. After this, all images of the biopsy were analyzed. Excluded were epithelium, submucosal glands, airway smooth muscle tissue and damaged tissue. Afterwards, the algorithm determined the immuno-positive area and the measured area of the biopsy, leading to the percentage positive area per biopsy. A positive area was at least 11.8 μm^2^, to exclude false positive areas. In this manner, the total positive area and the total measurable area of the biopsy were quantified and the percentage positive area per biopsy was calculated. The smallest evaluable area per section (after exclusion of epithelium, submucosal glands, airway smooth muscles and damaged tissue) was 0.4 mm^2^. The mean percentage positive area of two biopsies was used. Measurements were performed in a blinded way by 2 observers (B.R. and B.W.).

### Data analysis

Analyses were performed using SPSS for Windows 10.0 (SPSS Inc., Chicago, IL). Values of p < 0.05 were considered statistically significant. Clinical data were expressed in means (± SD) or geometric means (minimum-maximum); inflammatory data were expressed in medians (minimum-maximum). Differences between asymptomatic smokers, symptomatic smokers (GOLD 0) and smokers with COPD (GOLD stage I, II and III) were analysed using the Kruskall-Wallis test, a non-parametric equivalent to one-way ANOVA. Only, when the Kruskall-Wallis test was significant the Mann Whitney U test was used to analyse the differences between the 3 groups. Differences between GOLD stages 0, I, II, and III were analysed using the Kruskall-Wallis test, when this test was significant the Mann Whitney U test was used to analyse the differences between the different GOLD stages.

Correlations between smoking characteristics and lung function parameters were calculated with Pearson correlation test. Correlations between inflammatory cells and mediators in sputum and/or bronchial biopsies and smoking characteristics or lung function parameters were calculated with Spearman's rank correlation test. The subjects with GOLD stages I-III were used to investigate the correlations in COPD patients.

## Results

### Asymptomatic smokers versus smokers with GOLD stage 0-III

The 34 smokers with COPD were categorised into GOLD stage 0 'symptomatic smokers' (n = 9), GOLD stage I (n = 9), stage II (n = 10) and stage III (n = 6); none of the patients fulfilled the criteria for GOLD stage IV. The clinical characteristics of all subjects are presented in (see Additional file 1). Symptomatic smokers (GOLD stage 0) had significantly decreased lung function and more severe hyperresponsiveness to AMP than asymptomatic smokers had. COPD patients in GOLD stages I-III were older, had significantly more pack-years smoking, lower airway conductance and more severe hyperresponsiveness to AMP and methacholine than asymptomatic and symptomatic (GOLD 0) smokers.

#### Sputum

Two asymptomatic smokers could not produce sputum. The median (range) percentage non-squamous cells was 94 (75–99)% in COPD patients and 88 (64–99)% in asymptomatic smokers (table 1). Symptomatic smokers (GOLD 0) had higher percentage of sputum neutrophils than asymptomatic smokers. Smokers with COPD (GOLD stage I-III) had higher percentage of neutrophils, IL-8 and ECP levels in sputum than asymptomatic smokers, and higher IL-8 levels in sputum than symptomatic smokers. The percentage of macrophages was lower (table 1). In the separate GOLD stages, GOLD stage II had a higher percentage of sputum neutrophils compared with the asymptomatic smokers (70% and 60% respectively) and higher IL-8 and ECP levels in sputum than GOLD stage 0 and I (21.4 ng/ml versus 8.7 and 8.5 ng/ml respectively, and 291 μg/L versus 120 and 99 μg/L respectively). GOLD stage III had higher levels of IL-8 than GOLD stage 0 (27.7 ng/ml and 8.7 ng/ml respectively) and lower ECP levels than GOLD stage II (87 μ/L versus 291 μg/L).

**Table 1:** Sputum inflammation in smokers with COPD, symptomatic smokers and asymptomatic smokers

	**COPD**	**Symptomatic smokers**	**Asymptomatic smokers**
	**GOLD I-III**	**GOLD 0**	
*Sputum*, *n*	25	9	24
Volume, ml	4.1 (0.7–14.3)	3.1 (0.3–10.0)	2.3 (0.6–10.8)*
Total cells, 10^6^	6.7 (1.4–54.5)	4.1 (1.1–15.3)	3.5 (0.2–23)*
Cell conc., 10^3^/ml	1507 (484–9620)	2134 (534–4146)	1445 (303–4592)
Nonsquamous cells, %	94 (75–99.7)	92 (81–96)	88 (64–99.5)
Eosinophils, 10^3^/ml	15 (0–106)	20 (0–135)	13 (0–235)
%	1.4 (0–4.0)	1.8 (0–4.1)	0.8 (0–12.6)
Neutrophils, 10^3^/ml	870 (235–7608)	1575 (434–2558)	661 (164–2856)
%	72.6 (45–89)	66 (39–81)	60.1 (31.5–92.6)* †
Macrophages, 10^3^/ml	407 (89–2615)	535 (89–2422)	568 (22–1488)
%	25.4 (8.2–52.7)	28.7 (16.6–58.4)	36 (6.5–62.4)*
Lymphocytes, 10^3^/ml	14 (0–77)	15 (0–62)	11 (1–161)
%	0.8 (0.1–4)	0.8 (0.4–1.6)	0.7 (0.1–3.8)
Epithelial cells, 10^3^/ml	10 (0–107)	0.4 (0–84)	10 (0–55)
%	0.5 (0–11)	0.5 (0–2.5)	0.8 (0–6.6)
Basophils, 10^3^/ml	0 (0–4)	0 (0–12)	0 (0–8)
%	0 (0–0.1)	0 (0–0.3)	0 (0–0.3)
IL-8, ng/ml	16.8 (2.1–161)†	8.7 (0.1–25.7)	5.3 (0–25)*
ECP, μg/L	157 (32–2700)	119.8 (13.3–238)	66 (4.7–1282)*

Values expressed in median (range). Abbreviations: cell conc. = cell concentration; IL-8 = interleukin-8; ECP = eosinophilic cationic protein.

* p < 0.05 asymptomatic smokers *versus *total COPD (I-III), Mann-Whitney -U test, † p < 0.05 *versus *GOLD stage 0, Mann-Whitney-U test.

#### Bronchial biopsies

Bronchial biopsies could not be collected or were of insufficient quality in 2 asymptomatic smokers, in 1 subject GOLD stage 0, and in 6 COPD patients. The percentage positive area of inflammatory cells in bronchial biopsies (CD3, CD4, CD8, CD20, neutrophils, macrophages, eosinophils and mast cells) did not differ between COPD (GOLD I-III), symptomatic smokers (GOLD 0) and asymptomatic smokers (table 2). Only COPD patients with GOLD stage II had a higher percentage positive CD3 area than asymptomatic smokers (1.84 (0.24–9.24) and 0.76 (0.17–2.4) respectively).

**Table 2:** Inflammation in bronchial biopsies from smokers with COPD and asymptomatic smokers

	**Total COPD**	**Symptomatic smokers**	**Asymptomatic smokers**
	**GOLD I-III**	**GOLD 0**	
*Biopsies*, *n*	19	8	24
CD3, %positive area	1.05 (0.2–9.24)	0.68 (0.19–1.7)	0.76 (0.17–2.4)
CD4, %positive area	0.041 (0.01–0.57)	0.073 (0–0.18)	0.04 (0–0.15)
CD8, %positive area	0.27 (0.03–2.55)	0.19 (0.02–1.53)	0.33 (0.3–1.25)
CD4/CD8 ratio	0.19 (0.1–4.4)	0.39 (0.04–1.2)	0.18 (0–0.91)
CD20, %positive area	0.003 (0–3.40)	0.003 (0–0.23)	0.005 (0–0.12)
NP57, %positive area	0.025 (0–0.13)	0.05 (0–0.23)	0.021 (0–0.36)
CD68, %positive area	0.035 (0–0.21)	0.056 (0–0.16)	0.041 (0–0.32)
EG2, %positive area	0.021 (0–0.31)	0.049 (0–0.15)	0.063 (0–0.59)
AA1, %positive area	0.15 (0.01–0.91)	0.22 (0–0.41)	0.22 (0.1–1.16)

Values expressed in median (range). Abbreviations: CD20 = B-cell marker, NP57 = neutrophil elastase, CD68 = macrophages, EG2 = eosinophils, AA1 = mast cell tryptase.

### Correlations of lung function with smoking and airway inflammation

FEV_1 _post BD (% predicted) correlated negatively with the number of pack-years smoking (r = -0.51, p = 0.03) in COPD, but not significantly with the number of cigarettes smoked per day.

FEV_1 _post BD correlated negatively with IL-8 levels in sputum and positively with macrophages in sputum and mast cells in bronchial biopsies of patients with COPD (table 3). The latter correlation was mainly caused by 4 patients with low mast cell positive areas. In asymptomatic smokers, no significant correlations were found between lung function and airway inflammation (table 3).

**Table 3:** Spearman's rank correlations between current smoking and airway obstruction and airway inflammation.

	COPD GOLD I-III (n = 19)		Asymptomatic smokers (n = 26)	
	rho	p-value	rho	p-value
*Cigarettes/day*				
Neutrophils sputum, %	-0.46	0.02	-0.24	NS
Macrophages sputum, %	0.44	0.027	0.35	0.095
Eosinophils sputum, %	-0.19	NS	-0.39	0.057
Eosinophils sputum, 10^6^/ml	-0.11	NS	-0.44	0.029
CD68 biopsy, % pos. area	0.69	0.002	0.46	0.029
EG2 biopsy, % pos. area	-0.79	0.001	0.02	NS
*Pack-years smoking*				
CD68 biopsy, %pos. area	0.48	0.043	0.21	NS
*FEV_1 _post BD, %pred*.				
Neutrophils sputum, %	-0.31	NS	0.15	NS
Macrophages sputum, %	0.41	0.046	-0.22	NS
IL-8 sputum, ng/ml	-0.54	0.007	0.15	NS
CD68 biopsy, %pos. area	0.33	NS	0.18	NS
AA1 biopsy, %pos. area	0.53	0.02	0.09	NS

CD68 = macrophages; EG2 = eosinophils; % pos. area = percentage positive area; FEV1 = forced expiratory volume in one second; post BD = post bronchodilator (15 minutes after 400 μg salbutamol); IL-8 = interleukin 8; AA1 = mast cells; NS = not significant

AHR did not correlate with number of cigarettes smoked per day, number of pack-years smoking or airway inflammation in sputum or bronchial biopsies in both asymptomatic smokers and COPD patients (data partially presented and discussed earlier: Willemse et al, [35]).

### Correlations of current smoking with airway inflammation

**Figure 1:**
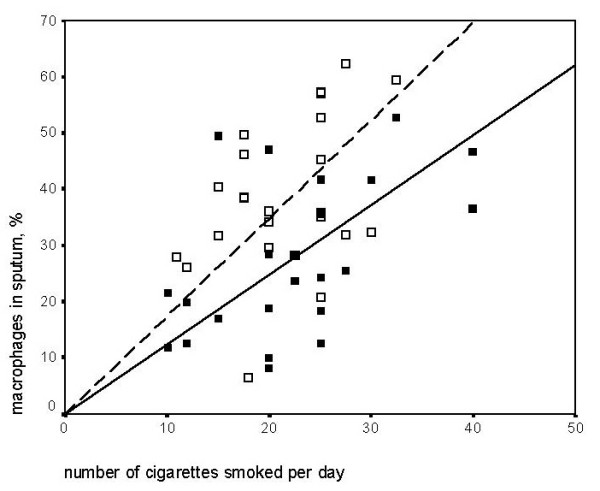
Spearman's rank correlation: Cigarettes smoked per day and macrophages in induced sputum. COPD (■, ): rho = 0.44 p = 0.03 and asymptomatic smokers(□, ): rho = 0.35 p = 0.1.

**Figure 2:**
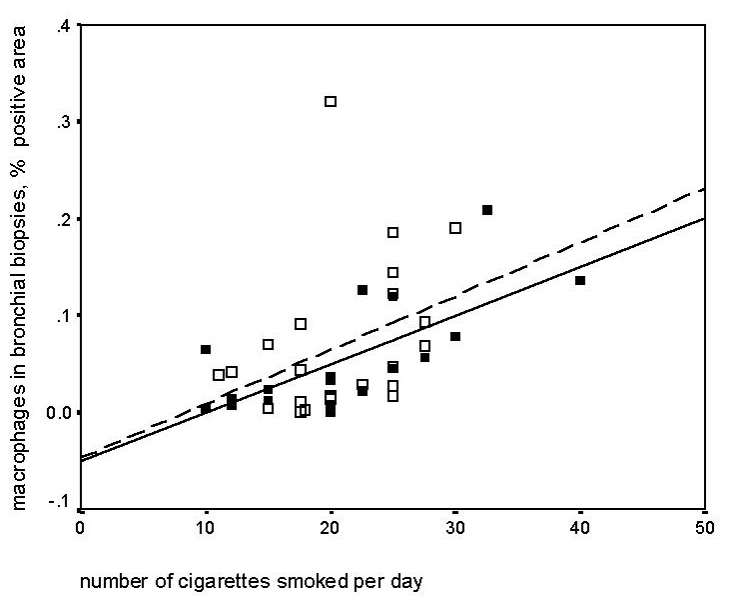
Spearman's rank correlation: Cigarettes smoked per day and percentage of macrophages in biopsies. COPD (■, ): rho = 0.69 p = 0.002 and asymptomatic smokers(□, ): rho = 0.46 p = 0.03.

The number of cigarettes smoked per day correlated negatively with neutrophils and positively with macrophages in sputum, which was significant in COPD (table 3, figure 1). The number of cigarettes smoked per day correlated positively with macrophages in bronchial biopsies, in both groups (table 3, figure 2). In asymptomatic smokers, the number of cigarettes per day correlated negatively with the number and percentage of eosinophils in sputum. In COPD the number of cigarettes smoked per day correlated negatively with eosinophil area in bronchial biopsies (table 3).

### Correlations of pack-years smoking with airway inflammation

In COPD patients pack-years smoking was positively correlated with the macrophage percentage positive area (table 3). Otherwise no significant correlations were found.

## Discussion

This study shows that asymptomatic smokers, symptomatic smokers (GOLD stage 0), and smoking patients with COPD have a large overlap in inflammation as assessed in sputum and airway wall biopsies. Patients with stage GOLD I-III had a higher percentage of neutrophils, and higher ECP and IL-8 levels in sputum than asymptomatic smokers, and higher IL-8 levels than symptomatic smokers. In symptomatic smokers percentage sputum neutrophils were higher than in asymptomatic smokers.

Whereas current smoking was associated with higher numbers of inflammatory cells in both asymptomatic smokers and COPD patients, pack-years smoking was only associated with higher airway wall macrophages in COPD and to the severity of airway obstruction. More severe airway obstruction in its turn was associated with lower percentage of sputum macrophages in smokers with COPD. Thus, the small difference in airway inflammation found between smokers with and without COPD may be due to the interference of current cigarette smoking.

This study demonstrates that a higher number of daily smoked cigarettes is associated with a higher percentage of macrophages in bronchial biopsies and sputum, both in smokers with COPD and asymptomatic smokers. In addition, eosinophils and neutrophils in sputum were negatively correlated to current smoking. Only few studies have provided data on correlations between airway inflammation and current smoking since smokers and ex-smokers were generally investigated together as one group. Two studies reported a positive correlation between neutrophils in bronchoalveolar lavage and the number of cigarettes smoked per day when asymptomatic smokers, chronic bronchitis patients and COPD patients were analysed together [7,13]. One study in asymptomatic smokers reported that the number of cigarettes smoked per day correlated positively with macrophages and IL-8 levels in bronchoalveolar lavage [36]. Macrophages in the central airways of smokers with and without COPD may be a direct inflammatory reflection of current smoking. On the other hand, it is not likely that current smoking is the only factor responsible for the accumulation of macrophages, since they are also increased in bronchial biopsies of ex-smokers with COPD [37]. Furthermore, we show that not only current smoking but also a higher number of pack-years smoking is associated with higher number of macrophages in COPD. This suggests that effects of current smoking are superimposed upon the underlying macrophage infiltration, which is part of the ongoing inflammatory process in COPD. This is important to realise when investigating the inflammatory and remodelling processes in smokers and ex-smokers with or without COPD. We therefore strongly suggest to avoid including mixed populations of smokers and ex-smokers in future studies on inflammatory processes in COPD

Current smoking was negatively related to eosinophils, i.e. the more cigarettes smoked per day the fewer eosinophils were present in sputum of asymptomatic smokers and in bronchial biopsies of patients with COPD. It may be that smoking has an anti-inflammatory effect on eosinophils or may influence cell kinetics. It has been suggested that carbon monoxide (CO) present in cigarette smoke has an anti-inflammatory potential [38,39], at least with respect to certain cell types and/or subsets. The extent and relevance of this supposed anti-inflammatory effect in humans remains to be established, but in guinea pigs it has been shown that acute cigarette smoke exposure suppresses the number of eosinophils after 6, 12 and 24 hours [40]. This may indicate that even the cigarettes smoked 24 hours before sputum induction or bronchoscopy could have induced this inverse relationship between current smoking and eosinophilic inflammation, since our participants refrained from smoking for 8 hours before the bronchoscopy. Nevertheless, it is well known that repetitive smoking for several years causes extensive damaging effects, indicating that the long-term overall effects of cigarette smoke dominate the anti-inflammatory effects.

Macrophages in bronchial biopsies of smokers with COPD were positively associated with pack-years smoking. No other relationships between pack-years smoking and airway inflammation were found in our study. This is in agreement with previous studies which either did not find any correlations [41] or did not investigate this [11,15,42]. Only Lams *et al*. [24] reported a positive correlation between CD8+ cells in bronchial biopsies and pack-years smoking and a negative correlation between neutrophils in bronchial biopsies and pack-years smoking, when all smokers (COPD and asymptomatic smokers) were analysed. In broncho-alveolar lavage percentage neutrophils was positively associated with pack-years smoking when all smokers and ex-smokers with and without COPD were analysed together [7,13].

One would expect that in COPD patients inflammatory markers would be more related to pack-years smoking instead of the number of cigarettes smoked per day. However, only macrophages in bronchial biopsies showed a positive correlation with pack-years smoking whereas macrophages, eosinophils and neutrophils were related to the number of cigarettes smoked. This may indicate that some of the inflammation due to cumulative smoke exposition is overruled by inflammation caused by current smoking. Neutrophils and eosinophils are "fast moving, or transient" inflammatory cells, whereas macrophages remain much longer in the lung tissue. This stresses the importance of macrophages in the development and progression of COPD.

This study shows that the percentage of neutrophils in sputum is higher in smokers with COPD (median 72.6%) than in asymptomatic smokers (median 60.1%), especially in GOLD stage II. This is completely in line with results of previous studies, which showed that smokers with moderate to severe COPD had higher total cell numbers and percentages of neutrophils in sputum than asymptomatic smokers [11,15,42]. Thus our finding suggests that this aspect of inflammation is associated with disease severity.

In symptomatic smokers (GOLD stage 0) the percentage of neutrophils in sputum was higher than in asymptomatic smokers, but similar to COPD patients. This has not been investigated in induced sputum before, however in broncho-alveolar lavage neutrophils show the same pattern [12]. No other differences were found in airway inflammation between symptomatic smokers and asymptomatic smokers. This is in contrast to the study of Sun et al [43], who investigated smokers with chronic bronchitis and found not only an increased number of neutrophils in broncho-alveolar lavage, but also increased eosinophils, mast-cells, CD4 positive and CD8 positive T cells compared to "healthy" smokers. This suggests that chronic bronchitis is better reflected by broncho-alveolar lavage than by induced sputum or bronchial biopsies.

In the present study, IL-8 levels in sputum were significantly higher in smokers with COPD than in asymptomatic and symptomatic smokers. In addition, higher IL-8 levels strongly correlated with more severe airway obstruction in smokers with COPD. This is in line with the data of Keatings *et al*. who showed that both IL-8 and percentage of neutrophils in sputum were increased in patients with moderate COPD as compared to asymptomatic smokers [15]. This may suggest that IL-8, a chemoattractant of neutrophils and an activator of MMP-9, plays a role in the development of airway obstruction. Alternatively, this may reflect the airway obstruction present.

Inflammatory cell density in bronchial biopsies did not significantly differ between smokers with COPD (GOLD I-III), asymptomatic smokers and symptomatic smokers. Only CD3 percentage positive areas in bronchial biopsies were higher in smokers with COPD stage II than in asymptomatic smokers. In agreement with our findings, other studies [24,41] investigating smokers with and without COPD, found no differences in neutrophils, macrophages, eosinophils, CD4 positive cells or CD4/CD8 ratio in bronchial biopsies. In contrast, one previous study demonstrated a higher number of CD8+ cells in smokers with predominantly moderate COPD compared to asymptomatic smokers [24]. In addition, two other studies demonstrated that CD3+ and CD8+ cell numbers were lower and macrophages and neutrophils were higher in smokers with severe COPD [22,41]. It may thus well be that differences between smokers with and without COPD become only apparent in case of severe COPD. Unfortunately the number of patients with evaluable biopsies was too small in our study population (n = 4) to investigate whether this indeed is the case.

A factor that should be taken into consideration is the age difference between the COPD patients and asymptomatic smokers under study. Previous studies investigated younger (mean age 35 years) asymptomatic smokers than our participants (mean age 50 years) [11,15,42]. The composition of sputum may differ between older and younger healthy subjects, as shown in bronchoalveolar lavage where the number of total cells and neutrophils increase with age [44]. Since we investigated COPD patients and asymptomatic smokers of almost similar age, our data are not hampered by age differences.

## Conclusion

Smoking COPD patients with GOLD stage I-III had almost similar airway wall and sputum inflammation as asymptomatic and symptomatic smokers without airway obstruction. Current smoking was associated with airway inflammation in patients with COPD and in asymptomatic smokers, whereas this was not the case for the cumulative pack-years smoked. In contrast, cumulative pack-years smoking was associated with the level of airway obstruction in COPD, suggesting that cumulative smoking induces chronic inflammation with subsequent sequelae of airway obstruction. Our results indicate that inflammatory effects of current smoking may mask findings of chronic inflammation in COPD, since numbers of inflammatory cells in bronchial biopsies and sputum are comparable in smokers with mild COPD and asymptomatic smokers.

## Authors' contributions

BW carried out the data collection and its coordination, immunohistochemical staining and quantification of the bronchial biopsies, performed the statistical analysis and interpretation of the data and drafted and revised the manuscript. NtH contributed to the conception and design of the study, the data collection and the interpretation of the data and revised the manuscript. BR carried out the sputum processing and immunoassays and revised the manuscript. DP contributed to the conception and design of the study, the data collection and the interpretation of the data and revised the manuscript. WT contributed to the conception and design of the study, the data collection and the interpretation of the data and revised the manuscript. All authors read and approved the final manuscript.

## Supplementary Material

Additional File 1 Description of the clinical characteristics of the participating subjects Click here for file
