# Supplementary material for: Association of current smoking with airway inflammation in chronic obstructive pulmonary disease and asymptomatic smokers
Source: Respir Res. 2005 Apr 25;6(1):38. doi: 10.1186/1465-9921-6-38 (PMC1140754; doi:10.1186/1465-9921-6-38)
Supplement: Additional File 1 — Description of the clinical characteristics of the participating subjects [file 1465-9921-6-38-S1.doc]

**Clinical c**haracteristics of participating subjects

|  | **COPD** | | | **Total COPD** | **Symptomatic smokers** | **Asymptomatic smokers** |
| --- | --- | --- | --- | --- | --- | --- |
|  | **GOLD I** | **GOLD II** | **GOLD III** | **GOLD I-III** | **GOLD 0** |  |
| N | 9 | 10 | 6 | 25 | 9 | 26 |
| Age, yr | 54 ± 5.6 | 60 ± 5.7^&,#^ | 56 ±4.9‡,^&^ | 57 ± 5.9* | 51 ± 3.6 | 50.4 ± 3.6 |
| Gender, M/F | 3/6 | 8/2 | 5/1 | 16/9 | 4/5 | 11/15 |
| Pack years, years | 35 ± 14.7 | 39 ± 9.0^&^ | 43 ± 12.9‡^&^ | 38 ± 12.1* | 28 ± 9.6 | 26 ± 8.0 |
| Cigarettes, number /day | 23 ± 8.0 | 22 ± 7.0 | 22 ± 10.3 | 22 ± 7.9 | 21 ± 6.1 | 21 ± 5.5 |
| FEV_1_ post BD, %pred. | 92 ± 8 | 70 ± 9^&, #^ | 42 ± 6.6‡^&,#,@^ | 70 ± 21* | 93 ± 15† | 109 ± 9.7 |
| FEV_1_/FVC post BD, % | 65 ± 3^&^ | 56 ± 7^&,#^ | 44 ± 6.3‡^&,#,@^ | 58 ± 9.6* | 78 ± 5 | 79 ± 4.7 |
| sGaw, 1/(kPa.s) | 1.1 ± 0.4 | 0.48 ± 0.23^&,#^ | 0.36 ± 0.09‡^&,#^, | 0.68 ± 0.4* | 1.2 ± 0.4† | 1.7 ± 0.7 |
| PC_20_ AMP, mg/ml | 34 (1.1-640) | 16 (1.9-640) ^&^ | 0.36 (0.02-4.4)‡^&,#,@^ | 13 (0.02-640)* | 108 (10-640) † | 286 (23.8-640) |
| PC_20_ Mch, mg/ml | 5 (0.9-78.2) | 1.5 (0.12-78.2) ^&^ | 0.20 (0.02-1.7)‡^&,#,@^ | 1.5 (0.02-78.4)* | 14 (0.9-78.2) | 25.2 (3.7-78.4) |

Values are expressed in means ± SD. PC_20_ AMP and PC_20_ MCh are expressed in geometric mean (range). M=male, F=female; sGaw= specific airway conductance; PC_20_AMP = provocative concentration of adenosine monophosphate causing a 20% fall in FEV_1_; PC_20_Mch = provocative concentration of methacholine causing a 20% fall in FEV_1_; post BD= post bronchodilator (15 minutes after 400 µg salbutamol). * p<0.05 total COPD (stage I-III) versus asymptomatic smokers / GOLD stage 0, Mann-Whitney-U test, † p<0.05 *versus* asymptomatic smokers, Mann-Whitney-U test, ‡ p<0.05 stage 0, stage I, stage II and stage III, Kruskall-Wallis test. & p<0.05 *versus* GOLD 0. # p<0.05 *versus* GOLD I. @ p<0.05 *versus* GOLD II.
